# Perigone Lobe Transcriptome Analysis Provides Insights into *Rafflesia cantleyi* Flower Development

**DOI:** 10.1371/journal.pone.0167958

**Published:** 2016-12-15

**Authors:** Xin-Wei Lee, Mohd-Noor Mat-Isa, Nur-Atiqah Mohd-Elias, Mohd Afiq Aizat-Juhari, Hoe-Han Goh, Paul H. Dear, Keng-See Chow, Jumaat Haji Adam, Rahmah Mohamed, Mohd Firdaus-Raih, Kiew-Lian Wan

**Affiliations:** 1 School of Biosciences and Biotechnology, Faculty of Science and Technology, Universiti Kebangsaan Malaysia, Bangi, Selangor, Malaysia; 2 Malaysia Genome Institute, Kajang, Selangor, Malaysia; 3 School of Environmental and Natural Resource Sciences, Faculty of Science and Technology, Universiti Kebangsaan Malaysia, Bangi, Selangor, Malaysia; 4 Institute of Systems Biology, Universiti Kebangsaan Malaysia, Bangi, Selangor, Malaysia; 5 Mote Research Limited, Babraham Hall, Babraham Research Campus, Cambridge, United Kingdom; 6 Genomics and Bioinformatics Centre, Malaysian Rubber Board, Rubber Research Institute of Malaysia, Sungai Buloh, Selangor, Malaysia; Youngstown State University, UNITED STATES

## Abstract

*Rafflesia* is a biologically enigmatic species that is very rare in occurrence and possesses an extraordinary morphology. This parasitic plant produces a gigantic flower up to one metre in diameter with no leaves, stem or roots. However, little is known about the floral biology of this species especially at the molecular level. In an effort to address this issue, we have generated and characterised the transcriptome of the *Rafflesia cantleyi* flower, and performed a comparison with the transcriptome of its floral bud to predict genes that are expressed and regulated during flower development. Approximately 40 million sequencing reads were generated and assembled *de novo* into 18,053 transcripts with an average length of 641 bp. Of these, more than 79% of the transcripts had significant matches to annotated sequences in the public protein database. A total of 11,756 and 7,891 transcripts were assigned to Gene Ontology categories and clusters of orthologous groups respectively. In addition, 6,019 transcripts could be mapped to 129 pathways in Kyoto Encyclopaedia of Genes and Genomes Pathway database. Digital abundance analysis identified 52 transcripts with very high expression in the flower transcriptome of *R*. *cantleyi*. Subsequently, analysis of differential expression between developing flower and the floral bud revealed a set of 105 transcripts with potential role in flower development. Our work presents a deep transcriptome resource analysis for the developing flower of *R*. *cantleyi*. Genes potentially involved in the growth and development of the *R*. *cantleyi* flower were identified and provide insights into biological processes that occur during flower development.

## Introduction

*Rafflesia* is a genus of holoparasitic flowering plants found only in the tropical rainforest of Southeast Asia. The genus is well known for members with extraordinary flower size and is deemed to represent the world’s largest single flower [1]. In contrast with conventional flowering plants, *Rafflesia* species are highly reduced in vegetative parts and the only visible component being the mycelium-like structure which penetrates their host plants [2]. Previous studies have reported on the absence of a plastid/chloroplast genome in *Rafflesia* [3]. This suggests that *Rafflesia* has lost its plastidial organelle, and it is therefore not surprising that this plant does not have the capability to carry out photosynthesis and thus depends completely on its host, the vines of the *Tetrastigma* tree species for nutrients and water supply. More interestingly, a recent study demonstrated that *Rafflesia* acquires benefits from its host through horizontal gene transfer mechanism from host to flower [4].

To date, more than 30 species of *Rafflesia* have been reported. Despite rapidly increasing discovery of new species, the biology of *Rafflesia* remains poorly understood. This may be mainly due to the limited resources available for research. The occurrence of *Rafflesia* is very rare as it takes a long time to complete its lifecycle [5]. The bud takes up to nine months to develop into a flower, but the flower lifespan is very short, as it blooms for only five to seven days before decomposing.

Molecular sequence information on *Rafflesia* is scarce. As a result, genome-driven elucidation of biological processes, particularly those that govern the growth and development of this giant flower plant remains under explored. In an effort to address this matter, we utilised next generation sequencing (NGS) technology to characterise the transcriptome of the perigone lobe (petal-like structure) of *Rafflesia cantleyi*. Sequence data were processed, assembled, annotated and classified into putative functional categories according to the Gene Ontology (GO) framework. The transcripts were further grouped into pathways based on the Kyoto Encyclopaedia of Genes and Genomes (KEGG) mapping. Transcripts from the developing flower were then compared to transcripts from a floral bud generated in a previous study [4] to identify genes involved in flower development. This is the deepest transcriptomic study to date of the developing flower of *R*. *cantleyi* and the results will serve as a reference for further gene expression and functional genomics studies.

## Materials and Methods

### Plant material and RNA isolation

**Fig 1:**
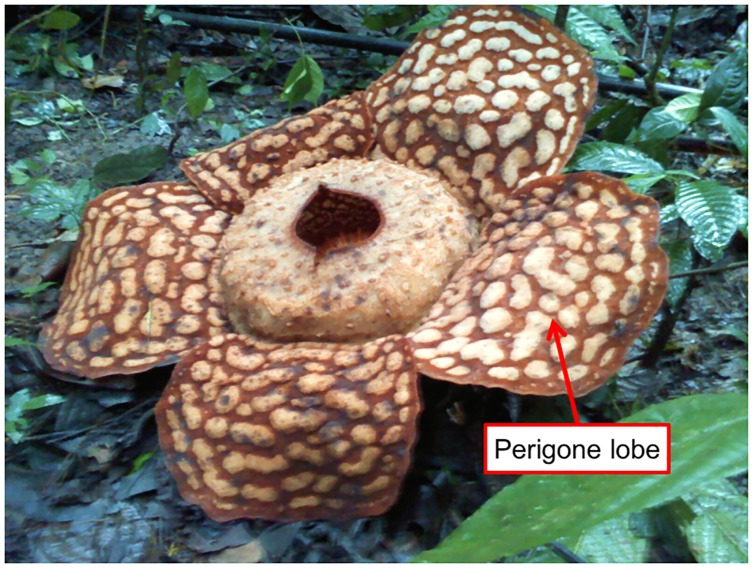
The *Rafflesia cantleyi* flower collected from Lata Jarum, Pahang, Malaysia.

The *R*. *cantleyi* flower (Fig 1) was collected from Lata Jarum, Pahang, Malaysia (Permission from Pahang State Forestry Department—reference no. PHN. PHG. (PEM) 118/146 Bhg. 3 (123)). The perigone lobe tissue was collected from the flower three days after it began blooming. One of the five perigone lobes present in the flower was dissected and transported back to the laboratory. Once in the laboratory, samples were fragmented into small pieces and stored at -80°C. Total RNA was extracted according to the method described by López-Gomez et al [6]. RNA integrity and quantity were quantified by Agilent BioAnalyzer (Agilent Technologies, USA) and Spectrophotometer ND-1000 (NanoDrop, USA).

### Sequencing and *de novo* assembly

A cDNA library was constructed with the TruSeq RNA Sample Prep kit (Illumina, USA) and sequenced on a HiSeq 2000 (Illumina, USA) with paired-end 90 bp read lengths. The reads were trimmed and filtered with FASTX-Toolkit to eliminate low quality reads. Reads were considered as high quality if more than 70% of the bases had phred values of more than Q20. Reads shorter than 30 bp after trimming were discarded from further analysis. Reads produced in this study have been deposited in the NCBI Sequence Read Archive (SRA) (accession number SRP075857). Three assembly tools, namely Oases [7], Trinity [8] and CLC Genomics Workbench (Version 4.9, CLC Bio), were evaluated for the *de novo* assembly of the *R*. *cantleyi* perigone lobe transcriptome. Preliminary assembled transcripts from each assembly tool were compared based on the number of transcripts generated, and average and total length of transcripts. The generated transcripts from each tool were then compared against the *Arabidopsis thaliana* proteome using BLASTX to determine the transcript set with the highest database hits by applying an *E*-value cut-off of 1E-06. Final assembly was carried out using Oases v0.2.08 (http://www.ebi.ac.uk/~/zerbino/oases/) [7]. The appropriate k-mer and coverage cut-off value used in the assembly was determined by a perl script (VelvetOptimiser-2.2.0.pl). K-mer size range from 25 to 79 were tested for the best N50 value while the coverage cut-off was automatically determined by the script. All other parameters were on default settings. Transcripts with a minimum length of 200bp obtained from final assembly were used for annotation and further analysis. This Transcriptome Shotgun Assembly project has been deposited at DDBJ/EMBL/GenBank under the accession GFBU00000000. The version described in this paper is the first version, GFBU01000000.

### Similarity search and functional annotation

To deduce the putative function, all transcripts were subjected to BLASTX analysis against the GenBank non-redundant protein database. Hits were considered as significant if *E*-value ≤ 1E-06. Gene ontology analysis was conducted on the annotated sequences through Blast2GO [9] followed by functional classification based on plant-specific GO slim ontology [10]. Putative metabolic pathway assignments were carried out according to KEGG mapping with the *E*-value cut-off of 1E-06. KOBAS (version 2.0.2) [11] was carried out in a stand-alone environment to identify enrichment in the KEGG pathway. To further characterise the transcriptome, we searched the annotated transcripts for possible functions involved in KOG classification. In addition, transcription factors represented in our samples were searched by performing BLASTX with an *E*-value cut-off of 1E-06 against transcription factor protein sequences obtained from the Plant Transcription Factor Database (PlantTFDB: http://planttfdb.cbi.pku.edu.cn/download.php) [12].

### Transcript abundance estimation

To analyse transcript abundance levels in the flower transcriptomes, Tophat [13] incorporating the Bowtie algorithm was used to align RNA-seq reads to the transcriptome. The aligned read files were processed by Cufflinks [14] to estimate the relative abundances of transcripts. The abundance of transcripts were upper-quartile normalised and corrected for sequence bias internally in Cufflinks. The unit of measurement for transcript abundance used is fragments per kilobase of exon per million fragments mapped (FPKM).

### Differential expression analysis

To analyse the difference of transcript abundance between the two floral development stages, which is bud and flower of *R*. *cantleyi*, the Cuffdiff algorithm was used. Cuffdiff allowed for the discovery of transcripts that are common, differentially expressed or present/absent between bud and flower tissues of *R*. *cantleyi*. Transcriptome data of floral bud used in this analysis was downloaded from NCBI SRA (http://www.ncbi.nlm.nih.gov/sra) (accession number: SRA052224) [4]. A reference transcriptome was built by performing a combine assembly of bud and flower RNA-seq reads using Oases. Tophat was used to align reads to the reference transcriptome while Cufflinks processed the alignment files produced by TopHat. Cuffmerge produces a combined GTF file, which is passed to Cuffdiff to re-estimate the abundance of transcripts listed in the GTF file and concurrently tests for differential expression. Genes with FDR < 0.05 were considered significant. A heatmap of selected differentially expressed genes was generated by CummeRbund (http://compbio.mit.edu/cummeRbund/).

### Reverse transcription quantitative real-time PCR (RT-qPCR)

For RT-qPCR analysis, eight candidate genes were selected from various levels of abundance. Actin was selected as the reference gene for relative quantification of gene expression. Gene-specific primers (S1 Table) were designed using Primer-Blast (https://www.ncbi.nlm.nih.gov/tools/primer-blast/). The primers pairs specificity and efficiency were examined using standard real-time PCR, and were further validated by gel electrophoresis to ensure correct amplicons. RT-qPCR was performed on DNase-treated RNA (RIN number 7.5; 20 ng/μl) in three technical replicates using QuantiNova^®^ SYBR^®^ Green RT-PCR Kit (Qiagen) following the manufacturer’s instructions. The amplification protocol: 10 min at 50°C and 2 min at 95°C for the reverse transcription, followed by 40 cycles of 5 s at 95°C and 10 s at 60°C using the CFX96^™^ Real-Time Detection System (Bio-Rad). The relative expression of each gene to actin was calculated from mean Ct ± standard error mean using the 2^-ΔCt^ method. Relative expression values from RNA-seq data were calculated from the ratio of FPKM value of individual genes relative to that of actin. Correlation analysis of relative expression values from RNA-seq and RT-qPCR was performed using Microsoft EXCEL 2016.

## Results and Discussion

### Sequence generation and *de novo* assembly

**Fig 2:**
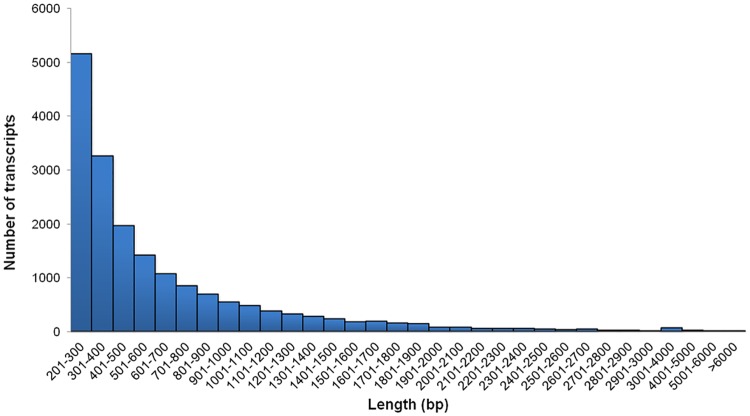
Length distribution of *Rafflesia cantleyi* flower transcripts obtained from *de novo* assembly.

Deep sequencing of transcriptome derived from the perigone lobe of *Rafflesia* was performed using the Illumina RNA-seq platform. More than 20 million paired-end reads with a length of 90 bp were obtained and subjected to filtering and trimming to remove low quality reads. After pre-processing, a total number of 40,768,187 high quality reads, corresponding to an average length of 87 bp were obtained. More than 99% of the raw reads were of quality more than Q20 (a sequencing error probability of 0.01), indicating the high quality of cDNA library and reads obtained. The summary of sequencing data is shown in Table 1. After cleaning and quality checks, the high quality reads were subjected to sequence assembly. In order to generate better assemblies, several assembly tools were evaluated for their performance, namely Oases, Trinity and CLC Genomics Workbench. Based on the number of transcripts, and average and total transcript length, Oases stood out as the best performer. Oases produced 38,783 transcripts with an average length of 1,172 bp, followed by Trinity (31,363 transcripts, average length of 865 bp) and CLC Genomics Workbench (26,294 transcripts, average length of 632 bp). Oases assembly also showed the largest significant similarity with *Arabidopsis* proteins (68.8%) compared to the transcripts generated by Trinity (55.6%) and CLC Genomics Workbench (44.9%) [Table 2]. Taken together, the results suggested Oases as the most suitable assembly tool for our dataset. As this study emphasises more on downstream analyses, the assembly output is crucial in determining the quality of the results. Thus, more stringent and conservative parameters were utilised during the assembly process to increase the confidence level of downstream analyses. A k-mer distribution generated by the Velvet perl script (S1 Fig) predicted that a k-mer size of 69 will produce the best N50 value, and was used in the final assembly process. In addition, a coverage cut-off of 4.86 was set to discard low-coverage transcripts. Subsequently, the high quality reads were assembled into 18,053 transcripts with a mean length of 641 bp. The size distribution of these transcripts is shown in Fig 2. Although most transcripts were between 200 and 300 bp in length, the assembly did produce a substantial number of longer contigs where 7,666 transcripts were more than 500 bp in length.

**Table 1:** Statistical summary of *Rafflesia cantleyi* sequence data.

Total number of paired-end reads (before trimming)	40,993,980
Total number of read base pairs (bp)	3,689,458,200
Average read length (before trimming; bp)	90
Total number of read paired-end (after trimming)	40,768,187
Total number of read singletons (after trimming)	306,421
Average read length (after trimming; bp)	87
Total number of reads assembled	33,002,956
Total number of transcripts produced	18,053
Average length of transcripts (bp)	641
Total number of transcript base pairs (bp)	11,572,001

**Table 2:** Comparison of *de novo* assembly data using Oases, Trinity and CLC Genomics Workbench.

	Oases	Trinity	CLC Genomics Workbench
Number of transcripts	38,783	31,363	26,294
Average transcript length (bp)	1172	865	632
Total transcript length (bp)	45,471,333	27,056,569	16,624,957
Annotated transcripts	26,672 (68.8%)	17,452 (55.6%)	11,807 (44.9%)

### Functional annotation

**Fig 3:**
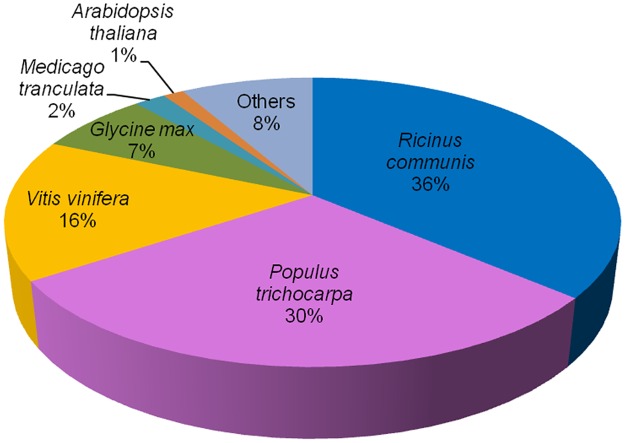
Species distribution of annotated *Rafflesia cantleyi* flower transcripts.

A comparison of the transcript sequences against the GenBank non-redundant protein database found a total of 14,336 (79.41%) transcripts with significant matches (S2 Table). The majority of these annotated transcripts displayed the highest similarity to genes from plants, with more than a third of the transcripts matched to *Ricinus communis*, partly due to the over representation of sequences from this species in the database and its close phylogenetic relationship with *R*. *cantleyi* (Fig 3).

**Fig 4:**
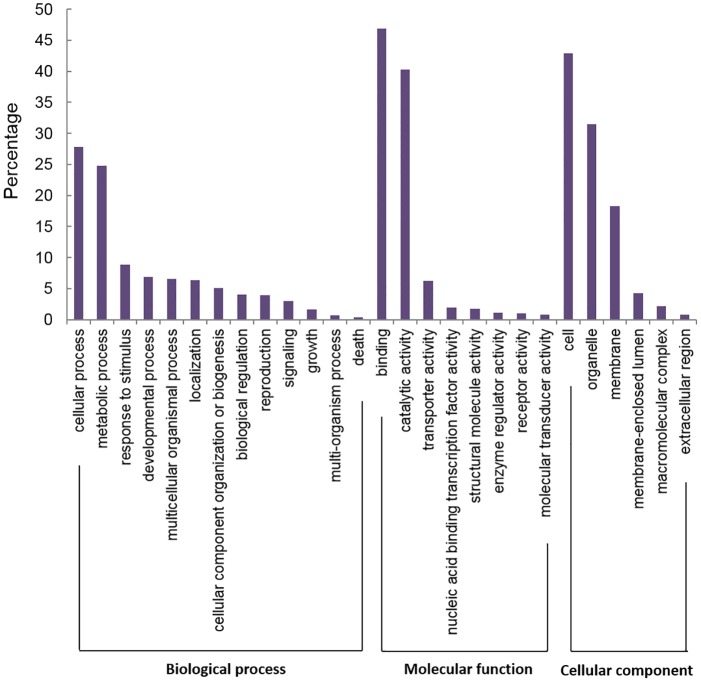
GO annotation of *Rafflesia cantleyi* flower transcripts.

All transcripts with BLAST hits were further classified using a set of plant specific GO slim terms. Information on protein domains were retrieved by InterProScan via Blast2GO and the corresponding annotations were merged with the annotated GOSlim terms. A total of 11,756 transcripts (65.1%) were annotated and classified to the three main categories: biological processes, molecular functions and cellular components (Fig 4). The biological process category was dominated by cellular (27.80%) and metabolic (24.74%) processes, which may be related to the developmental activities taking place during flowering. Besides that, a total of 930 transcripts were annotated with GOSlim terms related to post-embryonic development (GO: 0009791), 268 transcripts with flower development (GO: 0009908) and another 167 transcripts with pollination (GO: 0009856). The molecular function category was most highly represented by binding (46.89%) and catalytic activity (40.29%), while the cellular component category was dominated by cell (42.91%) and organelle (31.51%).

**Fig 5:**
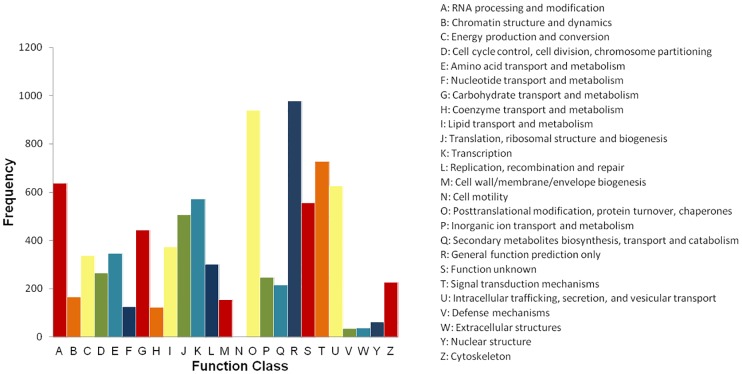
KOG functional classification of *Rafflesia cantleyi* flower transcripts.

Overall, 7,981 transcripts had KOG classification, which were distributed into 25 categories (Fig 5). Among these, the most represented group was “general function prediction only” (10.87%), followed by “post-translational modification, protein turnover, chaperone” (10.43%), “signal transduction mechanisms” (8.07%) and “RNA processing and modification” (7.24%). A total of 627 transcripts (6.96%) were in the intracellular trafficking, secretion, and vesicular transport category and 572 transcripts (6.35%) were categorised as having a role in transcription.

**Fig 6:**
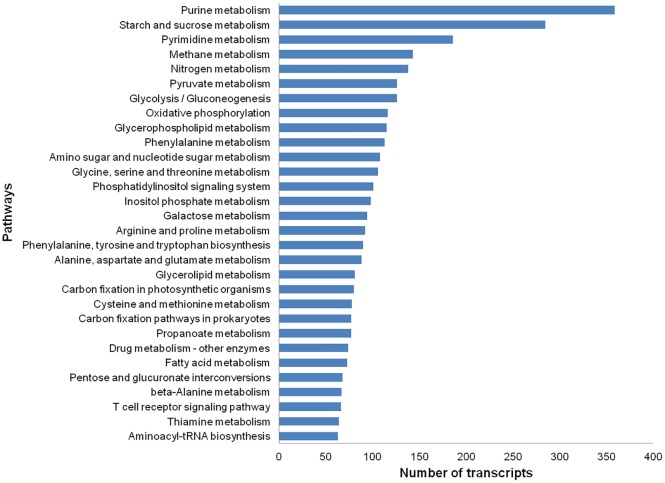
Top KEGG pathways represented in the *Rafflesia cantleyi* flower transcriptome.

We also retrieved KEGG pathways in which the transcripts were mapped by performing Enzyme Commission (EC) annotation via Blast2GO. In total, 2,537 transcripts were annotated with 627 ECs and were assigned to 129 different pathways (Fig 6; S3 Table). KOBAS analysis showed that the top five enriched pathways (FDR<0.05) were “metabolic pathways”, “biosynthesis of secondary metabolites”, “biosynthesis of amino acids”, “carbon metabolism” and “pyruvate metabolism” (S4 Table). A large assortment of pathways derived from this analysis showed that genes sampled from the *R*. *cantleyi* developing flower transcriptome represent a range of highly diverse functions. Notable dominant categories of metabolisms included carbohydrate metabolism (1322 transcripts), amino acid metabolism (860), lipid metabolism (620), and purine metabolism (545). Interestingly, a number of transcripts mapped to plant hormone signal transduction pathways that are known to regulate a variety of developmental processes such as senescence, stress response, and plant vegetative and reproductive growth. As shown in Table 3, such transcripts included those related to the signalling pathways mediated by ethylene, gibberellin, indole acetic acid, jasmonic acid, salicylic acid, auxin and cytokinin.

**Table 3:** The pathways and products involved in plant hormone signal transduction.

Pathway	Product	Pathway ID	Number of transcripts
Phenylalanine metabolism	Salicylic acid	Ko00360	113
Cysteine and methionine metabolism	Ethylene	Ko00270	78
Tryptophan metabolism	Auxin	Ko00380	57
α-Linolenic acid metabolism	Jasmonic acid	Ko00591	55
Indole alkaloid biosynthesis	Indole acetic acid	Ko00901	3
Diterpenoid biosynthesis	Gibberellin	Ko00904	2
Zeatin biosynthesis	Cytokinin	Ko00908	2

### Distribution of transcription factors

**Fig 7:**
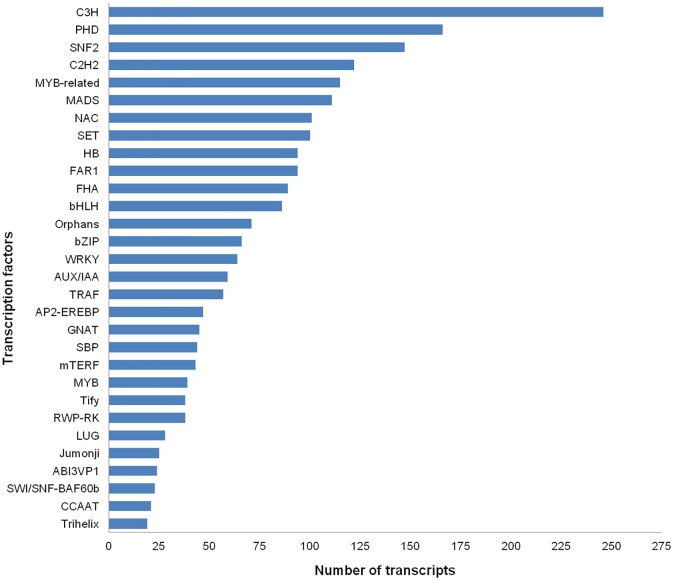
Transcription factor families identified in the *Rafflesia cantleyi* flower transcriptome.

Transcription factors are proteins consisting of various multigene families that bind to specific DNA sequences. In plants, the function of transcription factors are closely linked to gene expression and plant development. A total of 2,504 putative genes distributed in 75 transcription factor families were identified in the *R*. *cantleyi* developing flower transcriptome, representing 13.87% of the total transcripts (Fig 7; S5 Table). The majority of these annotated transcripts displayed the highest homology to the C3H family (246 transcripts, 9.82%). A total of 166 transcripts (6.63%) fell into the PHD family, followed by the SNF2 family (147 transcripts, 5.87%) and the C2H2 family (122 transcripts, 4.87%).

Previous studies have shown that one of the members of the C2H2 family, JAGGED (JAG) is expressed in the distal petal blade during late phase of development. It is believed to control petal growth and to promote cell-cycle progression and cell expansion [15–17]. In addition, quite a number of transcripts matched with transcription factors of the MADS family. MADS-box genes in plants have been intensively studied and many of the MADS family members have been shown to be involved in floral organ specification and development [18]. Several other transcription factor families associated with flower development were also found, including basic helix-loop-helix (bHLH) [19] and basic leucine zipper (bZIP) [20]. Previous work has demonstrated that mutations in the bHLH transcription factor, BIG PETAL (BPE) caused the failure of post-mitotic cell expansion and resulted in petal size change [21]. In short, the results obtained indicate that a diverse range of transcription factors is active in the *R*. *cantleyi* developing flower, consistent with what has been reported in floral development biology. Profiling of transcription factors will be a crucial starting point in elucidating *Rafflesia*-specific floral development.

### Transcript abundance analysis

**Fig 8:**
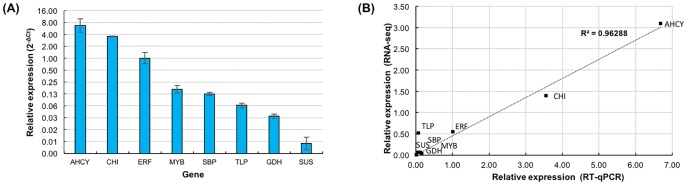
RT-qPCR validation of transcript abundance from RNA-seq analysis. (A) Relative expression of eight genes based on actin as the reference housekeeping gene. Y-axis is shown in log base 2 scale for clearer representation of genes with low expression values. (B) Correlation analysis of relative gene expression values from RT-qPCR and RNA-seq analysis. Genes studied include adenosylhomocysteinase (AHCY), chitinase (CHI), ethylene responsive transcription factor (ERF), MYB transcription factor (MYB), selenium binding protein (SBP), thaumatin-like protein (TLP), glutamate dehydrogenase (GDH) and sucrose synthase (SUS). The relative expression for RT-qPCR was calculated by using the 2^-ΔCt^ method based on the reference gene, actin; whereas the relative expression values for RNA-seq are FPKM ratios of individual genes relative to that of actin.

To analyse the abundance of *R*. *cantleyi* transcripts within the sample, quality-trimmed RNA-seq reads were mapped to the transcripts of *R*. *cantleyi*. The relative abundance of transcripts was calculated in fragments per kilobase of exon per million fragments mapped (FPKM) value. FPKM values (>0) for more than 76% of the transcripts were determined. The highest FPKM value is 17634.90 while the lowest FPKM value is 2.57, indicating a wide range of expression levels of *R*. *cantleyi* transcripts. Based on these FPKM values, transcripts were arbitrarily classified into five categories (very low, low, moderate, high and very high) (Table 4). The category with the largest fraction of transcripts (97.4%) showed low abundance (FPKM >10–500), followed by moderate (FPKM >500–1000) and very low (FPKM <10) abundance categories. Categories described as very low and very high each constituted less than 1% of the transcripts analysed (Table 4). To validate the transcript abundance, RT-qPCR was performed to quantify the relative expression of eight selected genes with different expression levels, i.e. two genes with a very high level of abundance, two genes with a moderate level of abundance, three genes with a low level of abundance and one gene with a very low level of abundance. The results showed a high correlation (R^2^>0.96) between the RNA-seq data and RT-qPCR (Fig 8).

**Table 4:** Distribution of transcripts according to the levels of abundance.

Levels of abundance	FPKM value	Number of transcripts	Percentage (%)
Very low	<10	98	0.71
Low	>10–500	13,421	97.40
Moderate	>500–1000	164	1.19
High	>1000–1500	50	0.36
Very high	>1500	52	0.38

For further analysis, we focused our analysis on selected genes in the highest abundance category (52 transcripts, 0.38%), which display functions that could provide insightful leads into *Rafflesia* floral development. Thirty-five out of the 52 transcripts that showed a very high abundance level had significant matches with protein sequences in the GenBank non-redundant database (S6 Table). The transcript with the highest FPKM value (17634.90) matched with the sufE-like protein of *Cucumis sativa*. Previous studies have suggested that the sufE-like protein in *Arabidopsis* (AtSufE) acts as an inter-organellar coordinator of Fe-S cluster biogenesis by interacting with the cysteine desulfurases (AtSufS) in plastids and AtNifS1 in mitochondria [22]. This ensures a balance of Fe-S cluster biogenesis between plastids and mitochondria, which is vital during embryogenesis. Three different SufE proteins, termed SufE1, SufE2 and SufE3 can be found in *Arabidopsis*. SufE1 is expressed in all tissues of *Arabidopsis* and its knockout is lethal [23, 24]. SufE2 was found to be abundantly expressed in the *Arabidopsis* flower, while SufE3 is ubiquitously expressed in all *Arabidopsis* organs [24]. In contrast to SufE1 and SufE2, SufE3 contains both a SufE-like domain and a domain similar to the bacterial quinolinate synthase NadA. SufE3 is considered as the quinolinate synthase enzyme of *Arabidopsis*, which is involved in a critical step during NAD biosynthesis. Our transcriptome showed the presence of both the SufE-like and quinolinate synthase domains. This may imply that the SufE-like protein found in our transcriptome displays a similar role with SufE3 in *Arabidopsis*, which also possesses both SufE and quinolinate synthase activities.

A putative homolog of fructose biphosphate aldolase (FBA) also showed a similar overrepresentation (FPKM value of 10157.30) in the flower transcriptome of *Rafflesia*. In plants, FBA is a key metabolic enzyme for glycolysis and gluconeogenesis [25]. It catalyses the condensation of fructose-1,6-biphosphate and sedoheptulose-1,7-biphosphate in the Calvin cycle [26]. It also plays important roles in sugar production, as well as abscisic acid (ABA) and stress signalling in plant. Previous study of eight FBA genes in *Arabidopsis* showed that the AtFBA gene family is greatly diversified in terms of subcellular and tissue localisation, and abiotic stress responsive expression patterns [27]. Studies conducted on these AtFBAs suggested that AtFBA1, AtFBA2, AtFBA5 and AtFBA7 were highly expressed in shoots, while AtFBA4 and AtFBA8 were found to have a higher expression in flowers. AtFBA3 and AtFBA6 have lower expression than the other AtFBAs but were shown to be specifically expressed in roots and flowers respectively. Moreover, AtFBA genes have been suggested to not only play important roles in development, but also in sugar, light and abiotic stress responses [27]. Given the significantly high levels of FBA transcripts in the *R*. *cantleyi* flower, we speculate that these genes may be involved in the flower development process, as in *Arabidopsis*.

Another transcript identified to be highly expressed in the *R*. *cantleyi* flower transcriptome is a putative homolog of the late embryogenesis abundant (LEA) gene (FPKM value of 4093.94). LEA proteins were first characterised in cotton and were abundantly found in the embryos at the late stage of seed development [28]. However, recent studies showed that LEA proteins in *Arabidopsis* are of a wide range of sequence diversity, intracellular localisation and expression patterns [29]. Besides that, LEA protein has been hypothesised to play a protective role in plant cells that might be essential for the survival of plant under various stress conditions [28, 30–32]. Many LEA genes can also be induced by ABA in both reproductive and vegetative tissues [29, 33, 34]. In general, expression of LEA proteins has been largely associated with water deficiency in plant tissues. The perigone lobes of *Rafflesia* have a high water content and water losses are at the highest level when the surface area is fully exposed during blooming. Thus, we suggest that the abundant LEA genes found in the *R*. *cantleyi* flower transcriptome confer a protective function towards water lost in perigone tissues. Accumulation of LEA proteins is believed to serve as a defence mechanism and inhibit excessive fluid loss.

### Detection of differentially expressed genes (DEGs)

**Fig 9:**
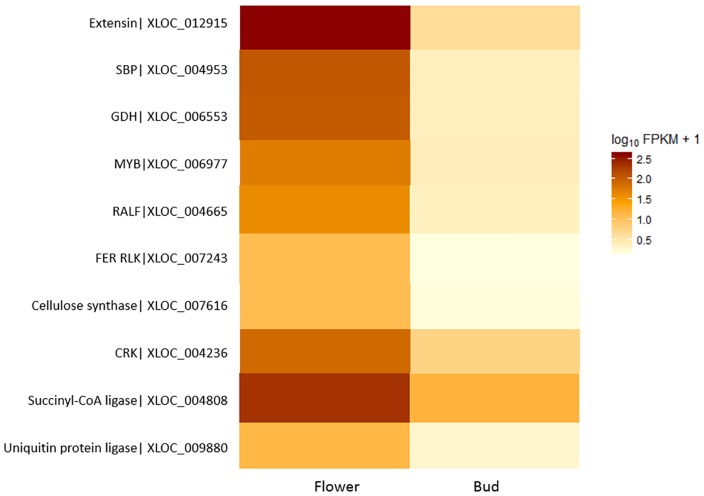
Heat map of selected genes that are differentially expressed between the developing flower and the floral bud of *Rafflesia cantleyi*.

Due to difficulty in obtaining *Rafflesia* material growing in the wild, this study was limited to transcriptome analysis of the perigone lobe. However, an existing floral bud transcriptome data [4] presented an opportunity to perform a preliminary comparative gene expression analysis of two floral development stages. One of the difficulties we had faced during this work was the absence of a suitable reference genome. Due to this, our strategy to quantify gene expression was to align RNA-seq reads back to the contigs that were reconstructed from the floral bud and developing flower transcriptome data sets. We had used Cuffdiff because it is more optimally designed to detect differential expression at the transcript level compared to the other DEG analysis tools. While edgeR has been reported to be able to detect more DEGs than Cuffdiff in many of previous comparative studies, it is more prone to introduce false positives. With the lack of reference data, we felt that it was more prudent to limit the introduction of false positives that we would not be able to filter or address appropriately at a downstream stage of the analysis. Furthermore, due to the limitation in getting biological replicates for DEG analysis, we had applied a more stringent gating to define DEGs. As Cuffdiff is more conservative in making differential expression calls, we think that it is more suitable for our study. Comparison of transcript abundance between the two transcriptome data sets revealed a total of 840 transcripts that were differentially expressed between the developing flower and the floral bud (S7 Table). Of these, 105 genes displayed higher expression in the perigone tissue of the flower compared to the floral bud, whereby 91 genes had GenBank matches (S7 and S8 Tables). To improve our understanding of *Rafflesia* floral development, we decided to examine several genes with functions related to stress response, cellular signalling, cell wall formation and transcriptional regulation. A heat map of these selected genes is shown in Fig 9.

Perigone lobes are petal-like structures of the *Rafflesia* flower. In angiosperms, petal development typically involves two stages, which is cell division and differentiation, followed by rapid growth through cell expansion [35, 36]. Differential analysis revealed that a number of genes associated with stress response were differentially expressed between the developing flower and the floral bud. This is not surprising as studies have shown that a large proportion of stress-related genes were also highly expressed in developing flowers of *Arabidopsis*, *Antirrhinum* and rose [37–39]. Such accumulation of stress-related proteins is believed to be induced by the rapid growth of petals and sudden increase in the rate of cellular metabolism [38]. Also, it has been suggested that petals are extremely sensitive toward stress conditions and therefore, abundant expression of stress response genes protects cells against intracellular or external stresses during flower development [39].

One of the transcripts encoding the stress-related gene, glutamate dehydrogenase (GDH) (XLOC_006553) showed more than 6-fold higher expression in the *R*. *cantleyi* flower in relation to the floral bud (S7 Table). GDH is known to catalyse the reversible reaction of glutamate and 2-oxyglutarate, which is a key reaction in carbon and nitrogen metabolisms in plants [40]. Previous studies of GDH in the tobacco plant showed that the gene was up-regulated by elevated ammonium levels, suggesting that it functions in ammonium detoxification by assimilating some of the ammonium ions [41]. Several other studies also reported the function of GDH in responding to abiotic and physiological stress conditions [42, 43]. Furthermore, GDH was found to localised in the cytosol of senescing organs, implying that it plays a role in controlling plant growth and productivity [44]. Taken together, we suggest that GDH plays an important role in enabling *R*. *cantleyi* developing flower cells to adapt or tolerate stress conditions during the rapid growth of perigone tissues.

Another differently expressed stress-related gene identified is the selenium binding protein (SBP) (XLOC_004953), which showed more than 6-fold higher expression in the *R*. *cantleyi* flower compared to the floral bud. High expression of SBP in *Oryza sativa* has been reported to enhance tolerance against pathogens [45]. In *Arabidopsis*, SBP was suggested to counter metal-related stresses and take part in the cadmium detoxification process [46, 47]. In addition, SBP has been reported to express ubiquitously in non-stress conditions especially in actively growing tissues and during seed development, highlighting its possible role in plant development [46]. Besides that, gene encoding succinyl-CoA ligase (XLOC_004808) and ubiquitin protein ligase (XLOC_009880) were also identified as up-regulated stress-related genes in the *R*. *cantleyi* flower.

Several genes involved in cellular signalling were differentially expressed between the *R*. *cantleyi* flower and floral bud. For example, XLOC_004665 that codes for a putative rapid alkalinisation factor (RALF) precursor showed a 4-fold increase of expression in the flower compared to the bud. RALF was first isolated from tobacco leaves and previous studies have shown that it arrested the growth of roots and pollen tubes in *Nicotiana attenuate*, *Arabidopsis* and tomato (*Solanum lycopersicum*) [48–50]. Besides that, there is evidence showing that RALF is a regulator of nodulation in *M*. *truncatula* [51]. Characterisation of five RALF-like genes in *Solanum chacoense* has also revealed the role of RALF proteins in plant development [52]. Increased expression of the RALF precursor gene in the *R*. *cantleyi* flower indicates that it plays an important role in regulating flower development.

Receptor-like kinase (RLK) proteins are known to play important roles in signal transduction pathways [53]. Several genes encoding RLKs were also identified to be differentially expressed in *R*. *cantleyi*, including XLOC_007243 (4.35-fold increase) and XLOC_004236 (3.97-fold increase), which are homologs of FERONIA (FER) receptor-like kinase and cysteine-rich receptor-like protein kinase, respectively. FER was previously revealed to act as a regulator for female fertility and mediating pollen tube rupture in the female gametophyte of *Arabidopsis* [54, 55]. It was also found to regulate RHO GTPase signalling of root hair development. Taken together, these findings indicate that FER plays specific growth and development roles in *R*. *cantleyi*.

Morphological differences between a developing flower and floral bud of *Rafflesia* is extremely pronounced, particularly in the extent of petal formation. As cell expansion in plants is highly dependent on cell wall structure and composition, genes encoding cell wall proteins and enzymes that are involved in such modifications would be differentially expressed during petal development [38]. Thus, this supports our observation of higher expression of cell wall associated genes in perigone tissue of the developing flower. Two such genes are extensin (XLOC_012915; 6.87-fold increase) and cellulose synthase (XLOC_007616; 4.12-fold increase). In *Arabidopsis*, expression of extensin has been detected in roots and inflorescences, and at regions of abscission and senescence [56]. Cellulose synthase is an enzyme involved in the synthesis of cellulose, which is the main component of plant cell walls [57].

Classes of transcription factors found in the developing flower transcriptome were described in a preceding section. Regulation of gene expression by transcription factors is instrumental during changes in plant development. XLOC_006977, which codes for a MYB transcription factor showed a greater than 5-fold increase in expression in the developing flower compared to the bud. In *Antirrhinum majuspetals*, there is evidence for control of different aspects of petal epidermal cell shape and subsequently overall petal presentation by a MYB-related transcription factor [58]. Homologs of this gene have also been identified in *Arabidopsis* and *Petunia hybrid* [59]. Changes in the shape of petal epidermal cells are believed to affect the perceived intensity of the colour signal that is important to attract pollinators. SEPALLATA (SEP) MADS box transcription factor was also identified in this study (XLOC_005948) and it showed a greater than 4-fold increase in gene expression in the flower compared to the bud. SEP appears to act in conjunction with another two MADS box transcription factors, namely APETALA3 (AP3) and PISTILLATA (PI), to regulate petal-specific differentiation [60].

## Conclusions

The biology of *Rafflesia* flower development is fascinating yet elusive. This is largely due to challenges in collecting ample and suitable material in the wild, lack of analytical methodologies developed for this plant species and extremely limited molecular resources. This study is an effort to apply a transcriptomic approach to profile genes expressed in *R*. *cantleyi*, particularly those that are abundantly expressed in the developing flower. A total of 18,053 transcripts were assembled from RNA sequencing of perigone tissues, with close to 80% assigned putative functions. This data set forms the first in-depth sequence resource in *Rafflesia* from which we have highlighted important genes that could provide clues to elucidating *Rafflesia* flower development. In future, characterisation of transcriptomes of sequential developmental stages from bud to mature flower will provide valuable information to shed light on its species-specific gene functions and unique floral biology.

## Supporting Information

S1 Fig K-mer distribution plot. (TIF)

S1 Table List of *Rafflesia cantleyi* genes selected for RT-qPCR analysis. (XLSX)

S2 Table List of annotation for *Rafflesia cantleyi* flower transcripts. (XLSX)

S3 Table List of enzyme code annotation for *Rafflesia cantleyi* flower transcripts. (XLSX)

S4 Table Top enriched KEGG pathways represented in the *Rafflesia cantleyi* flower transcripts. (XLSX)

S5 Table List of transcription factor annotation for *Rafflesia cantleyi* flower transcripts. (XLSX)

S6 Table Functional annotation of the 52 transcripts that showed very high abundance in the *Rafflesia cantleyi* flower transcriptome. (XLSX)

S7 Table Genes differentially expressed between flower and floral bud of *Rafflesia cantleyi*. (XLSX)

S8 Table Annotation of genes that showed higher expression in the perigone tissue of the flower compared to the floral bud of *Rafflesia cantleyi*. (XLSX)
