# Supplementary figures and images for: Perigone Lobe Transcriptome Analysis Provides Insights into Rafflesia cantleyi Flower Development
Source: PLoS One. 2016 Dec 15;11(12):e0167958. doi: 10.1371/journal.pone.0167958 (PMC5158018; doi:10.1371/journal.pone.0167958)

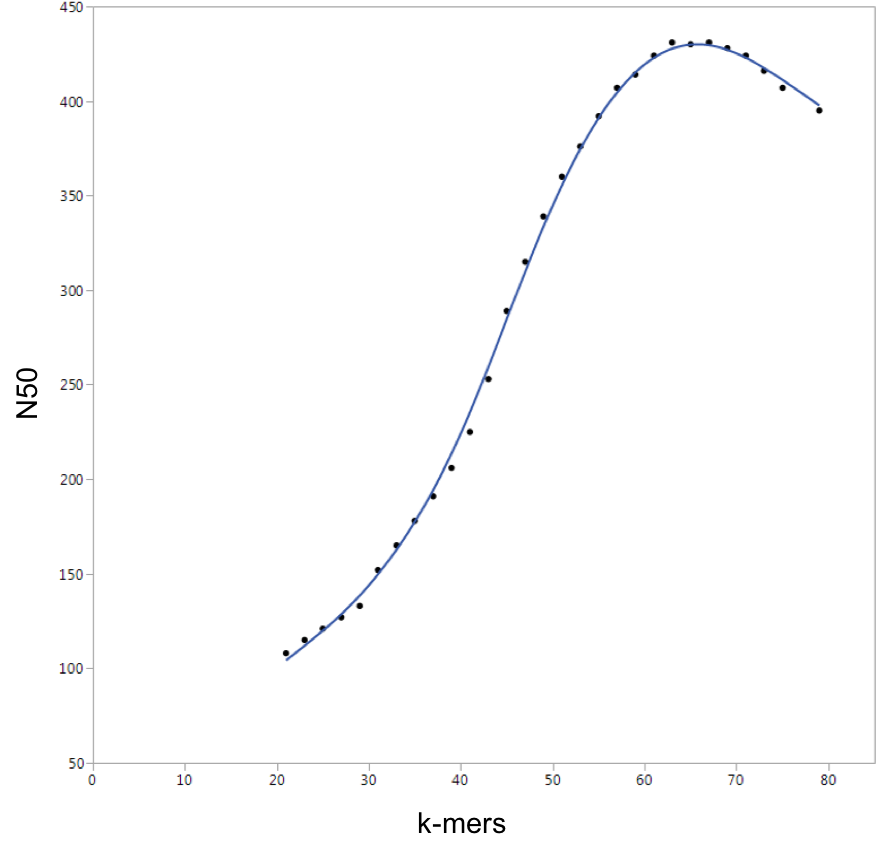

Supplement: S1 Fig — (TIF) [file pone.0167958.s001.tif]
